# Dietary Zinc Is a Key Environmental Modifier in the Progression of IgA Nephropathy

**DOI:** 10.1371/journal.pone.0090558

**Published:** 2014-02-28

**Authors:** Masayuki Maiguma, Yusuke Suzuki, Hitoshi Suzuki, Keiko Okazaki, Masashi Aizawa, Masahiro Muto, Yasuhiko Tomino

**Affiliations:** Division of Nephrology, Department of Internal Medicine, Juntendo University Faculty of Medicine, Tokyo, Japan; McGill University, Canada

## Abstract

IgA nephropathy (IgAN) shows diverse epidemiological characteristics, resulting from both genetic and acquired (e.g., environmental) causes. Environmental factors, such as diet or exposure to exogenous antigens, may prescribe the progression or prognosis of IgAN. It remains unclear as to how diet and infection influence susceptibility to IgAN. A relationship, such as Toll-like receptors (TLRs), especially TLR9 and TLR4, was demonstrated between IgAN and pathogen-recognition molecules. Recently, zinc (Zn) was discovered to be involved in various immune-related diseases, affecting B, T, and dendritic cells (DCs). This study investigates the relationship between dietary Zn and IgAN development in IgAN-prone mice. Seven-week-old IgAN-prone mice were divided into low, normal, and high Zn diet groups. To assess exogenous pathogen-mediated immune responses, lipopolysaccharide (LPS) was nasally administered. The activity of IgAN was biochemically and pathologically evaluated during the disease course. We also examined *in vitro* IgA production in spleen cells or in combinations of cocultured B, T, and DCs under various Zn conditions with or without LPS. Dietary conditioning with Zn affected serum immunoglobulins and urinary albumin levels, and mesangial deposition of IgA and IgG. Zn deficiency is associated with IgAN progression through the activation of the TLR4/TIR-domain-containing adapter-inducing interferon-β (TRIF), but not the TLR9, in DCs. Zn supplementation prevented disease aggravation. Our findings indicate that immune conditioning with dietary Zn alters nephritogenic IgA production after mucosal infection.

## Introduction

First described by Berger et al. in 1968 [1], IgA nephropathy (IgAN) is the most common primary chronic glomerulonephritis worldwide. Many factors, including mucosal infection [2], [3], genetic predisposition [4], diet [5], and hygiene [6], have been implicated in IgAN progression. Johnson et al. [7] suggested that environmental factors, such as exposure to antigens, affect the immune system and explain the difference in IgAN prevalence between developing and industrial countries. However, despite long-term research, the precise mechanism by which environmental factors affect IgAN severity is poorly understood.

The pathological impact of mucosal infection in IgAN has been established, as the disease is frequently exacerbated by upper respiratory or gastrointestinal infections. A series of studies are underway focusing on Toll-like receptors (TLRs), which are evolutionarily conserved regulators of the innate immune response. TLR activation may represent the final common pathway for exogenous antigens, which have a negative effect on the mucosa of patients with IgAN. We have reported that IgAN severity correlates with splenic TLR9 expression in IgAN-prone mice [8]. In addition, nasal challenge with CpG DNA (a ligand of TLR9) exacerbated glomerular damage and was accompanied by increases in serum IgA concentration and mesangial IgA deposition in these mice. This suggested that mucosal stimulation of TLR may be linked, in part, to production of nephritogenic IgA. In patients with IgAN, the expression of tonsillar TLR9 and TLR9 single nucleotide polymorphisms was correlated with the efficacy of tonsillectomy and steroid pulse therapy [9]. Coppo et al. [10] reported a significant correlation between TLR4 expression on circulating mononuclear (CD14+) cells and the levels of proteinuria and the phases of clinical activity in patients with IgAN. Accordingly, TLR-mediated innate immunity may be significantly involved in IgAN progression.

On the other hand, the prevalence of IgAN markedly differs depending on geographic location, suggesting the importance of diet and socioeconomic status. Donadio et al. [11] proposed that dietary supplementation with fish oils could benefit patients with immune-related renal diseases, including IgAN, lupus nephritis, and cyclosporine-induced nephrotoxicity. Coppo et al. [5] reported the influence of dietary gluten on primary IgAN. However, the alimentary effects on IgAN progression remain unclear.

An alimentary zinc (Zn) plays an important role in the functioning of the immune system [12]. Zn is a non-redox active ion essential for cell growth, development, and differentiation. In addition to its involvement with liver disease [13], growth retardation, and cognitive impairment, a Zn deficiency has many other negative effects [14], [15]. Emerging data [16]–[18] revealed that Zn deficiency in humans was correlated with an increased susceptibility to bacterial and/or viral infections, suggesting that Zn is the one of the most important alimentary factors for immune responses. Indeed, patients with Zn deficiency show defective cellular immunity, lymphopenia, and abnormalities in hematopoietic cells, including T cells [19], natural killer cells [20], and monocytes [21]. Stimulation with the TLR4 agonist LPS altered the expression of Zn transporters in the dendritic cells (DCs), thereby decreasing free intracellular Zn levels. A Zn chelator mimicked the effects of LPS, whereas Zn supplementation or overexpression of the gene encoding Zip6, a Zn transporter whose expression is reduced by LPS, inhibited LPS-induced upregulation of the Class II major histocompatibility complex and costimulatory molecules [22]. These results suggest a functional linkage between TLR signaling and Zn homeostasis in DCs. Ultimately, Zn deficiency may be involved in the pathogenesis of immune diseases via inappropriate immunological responses. In fact, Zn deficiency is often observed in patients with autoimmune diseases [16]. Accordingly, we hypothesized that dietary Zn levels are associated with susceptibility to IgAN via the modulation of the innate immune response in the mucosa, involving nephritogenic IgA production. In the present study, we assessed this hypothesis in IgAN-prone mice.

## Materials and Methods

### Mice

The “grouped ddY” (gddY) mice were established by selective mating of early-onset ddY mice for more than 20 generations. This resulted in a 100% incidence of severe disease at a young age [8], [23]–[25]. The mice were maintained in an SPF room at the animal facility of Juntendo University, Tokyo, Japan. The experimental protocol of this study was approved by the Ethics Review Committee for Animal Experimentation of Juntendo University Faculty of Medicine.

### Experimental Design

The basal mouse diet included the original formulation (AIN-93G) recommended by the American Institute of Nutrition for rodent feeding studies [26]. Basal diets were amended to yield three experimental diets containing the following (per kg):

- low Zn diet: 0 mg Zn;
- normal Zn diet: 67 mg Zn; and
- high Zn diet: 1000 mg Zn.

All diets were obtained from Oriental Yeast, Tokyo, Japan.

Female gddY mice at 7 weeks were randomly divided into three groups: low, normal, and high Zn diet groups. To assess the exogenous pathogen-mediated immune response, 100 ng LPS (055:B5; Sigma, USA) or saline as a vehicle control was nasally administered to mice from each group four times every 12 h before sacrifice. A histological examination was performed on each of the 10 gddY mice at 13 weeks of age. Blood samples were obtained from the buccal vein at 7, 9, and 11 weeks of age. Serum IgA, IgG, IgM, and IgA–IgG IC concentrations were measured using a sandwich enzyme-linked immunosorbent assay (ELISA) kit (Bethyl Laboratories). Urinary samples were obtained once a week from 7 weeks of age and stored at −80°C until analysis. Urinary albumin and creatinine levels were measured by immunoassay (DCA 2000 system; Siemens Healthcare Diagnostics, Tokyo, Japan) [25].

### Immunofluorescence

Kidneys were obtained after perfusion with normal saline. Renal specimens were mounted in an optimal cutting temperature (OCT) compound (Sakura Finetek, Tokyo, Japan) and stored at −80°C. Specimens embedded in the OCT compound were cut into 3-µm sections and fixed with acetone at −20°C for 5 min. IgA and IgG were stained by immunofluorescent staining. In brief, the sections were washed with phosphate-buffered saline (PBS), blocked with a blocking agent (DS Pharma Biomedical, Osaka, Japan) at room temperature for 30 min, and incubated with DyLight 488-conjugated goat anti-murine IgA (Bethyl laboratories, Texas, USA) and Alexa 555-conjugated goat anti-murine IgG (Invitrogen AG, Basel, Switzerland) antibodies at room temperature for 120 min. After three washing with PBS, the slides were mounted with a mounting medium (Dako, Tokyo, Japan). Samples were analyzed and imaged using confocal laser microscopy (Olympus Corporation, Tokyo, Japan). For the semi-quantitative analysis of the glomerular deposition of IgA or IgG, more than 10 glomeruli/cross section were observed and the following scores were assigned: 1 point, no glomerular deposition; 2 points, trivial glomerular deposition; 3 points, mild glomerular deposition; 4 points, moderate glomerular deposition; 5 points, severe glomerular deposition. The deposition score was calculated as follows:
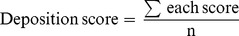



### Culture of Spleen Cells

The spleen was enucleated under aseptic conditions. Spleen cell suspensions were prepared by compression with the handle of a syringe in a Roswell Park Memorial Institute (RPMI) 1640 medium, followed by passage through a 100-µm nylon mesh. Red blood cells were removed by incubation with a Red Blood Cell Lysing Buffer (R7757; Sigma, USA) at room temperature for 1 min, followed by washing in a RPMI-1640 medium [27]. B cells, T cells, and DCs were isolated from the spleens of gddY mice using CD19 MicroBeads, a Pan T Cell Isolation Kit, and Pan DC MicroBeads (MACS; Milteny Biotec, Bergisch Gladbach, Germany) according to the manufacturer’s instructions. We used N,N,N′,N′-tetrakis (2-pyridylmethyl) ethylenediamine (TPEN) [22], a Zn chelator, to create low Zn conditions, and ZnSO_4_ with pyrithione to create high Zn conditions [22]. LPS (055:B5) and TPEN,2,2′-dithiodipyridine (D-5767) were obtained from Sigma. Pyrithione (2-mercaptopyridine N-oxide; P-24193) was obtained from Invitrogen. An RPMI 1640 medium with 5 µM TPEN was used for the spleen cell culture under low Zn conditions [22]. An RPMI 1640 medium with 5 µM ZnSO_4_ and 3 µM pyrithione was used for spleen cell culture under high Zn conditions [22]. IgA production by individual combinations of spleen cells was measured by ELISA after 48 h incubation. To evaluate the viability of B cells in each Zn condition, we cultured splenic B cells with anti-CD40 antibody (553721; BD Pharmingen, Tokyo, Japan) and anti-IgM antibody (115-006-020; Jackson ImmunoResearch Laboratories, Pennsylvania, USA) and then used the Cell Counting Kit-8 (Dojindo Laboratories, Tokyo, Japan) to quantify the cells.

### Real-time Reverse Transcription Polymerase Chain Reaction

RNA from spleen cells was purified with an RNeasy Mini Kit (74106; Qiagen, USA) after Zn conditioning or LPS stimulation. A SYBR Green PCR Master Mix (Applied Biosystems) and a 7500 Real-Time PCR System (Applied Biosystems) were used for the quantitative polymerase chain reaction. Transcript levels were normalized to those of the glyceraldehyde-3-phosphate dehydrogenase (Gapdh) gene. The primer sequences used were as follows: mouse TLR4, TGGCACTGTTCTTCTCCTGCCT and GGAATGTCATCAGGGACTTTGCTGA; mouse TLR9, TCTCCCAACATGGTTCTCCGTCG and TGCAGTCCAGGCCATGA; mouse MyD88, GCACCTGTGTCTGGTCCATT and CTGTTGGACACCTGGAGACA; mouse TRIF, TCTCTCCTCCTCCTCCAGCA and CGTCCCCTGCTGCAAAGAT; mouse interferon-β, GTCCTCAACTGCTCTCCACT and CCAGGCGTAGCTGTTGTACT; and mouse GAPDH, CATTGTGGAAGGGCTCATGA and TCTTCTGGGTGGCAGTGATG.

### Statistical Analysis

The correlation between different parameters was analyzed using analysis of variance. Data are expressed as the mean ± standard deviation or median values. p values <0.05 were considered statistically significant. All statistical analyses were performed using GraphPad Prism version 6.0 for Windows (GraphPad Software, San Diego, CA).

## Results

### Effect of Dietary Zinc in the IgAN Model Mice

We divided IgAN-prone gddY mice into three dietary groups (low, normal, and high Zn diet groups) and assessed IgAN progression.

**Figure 1:**
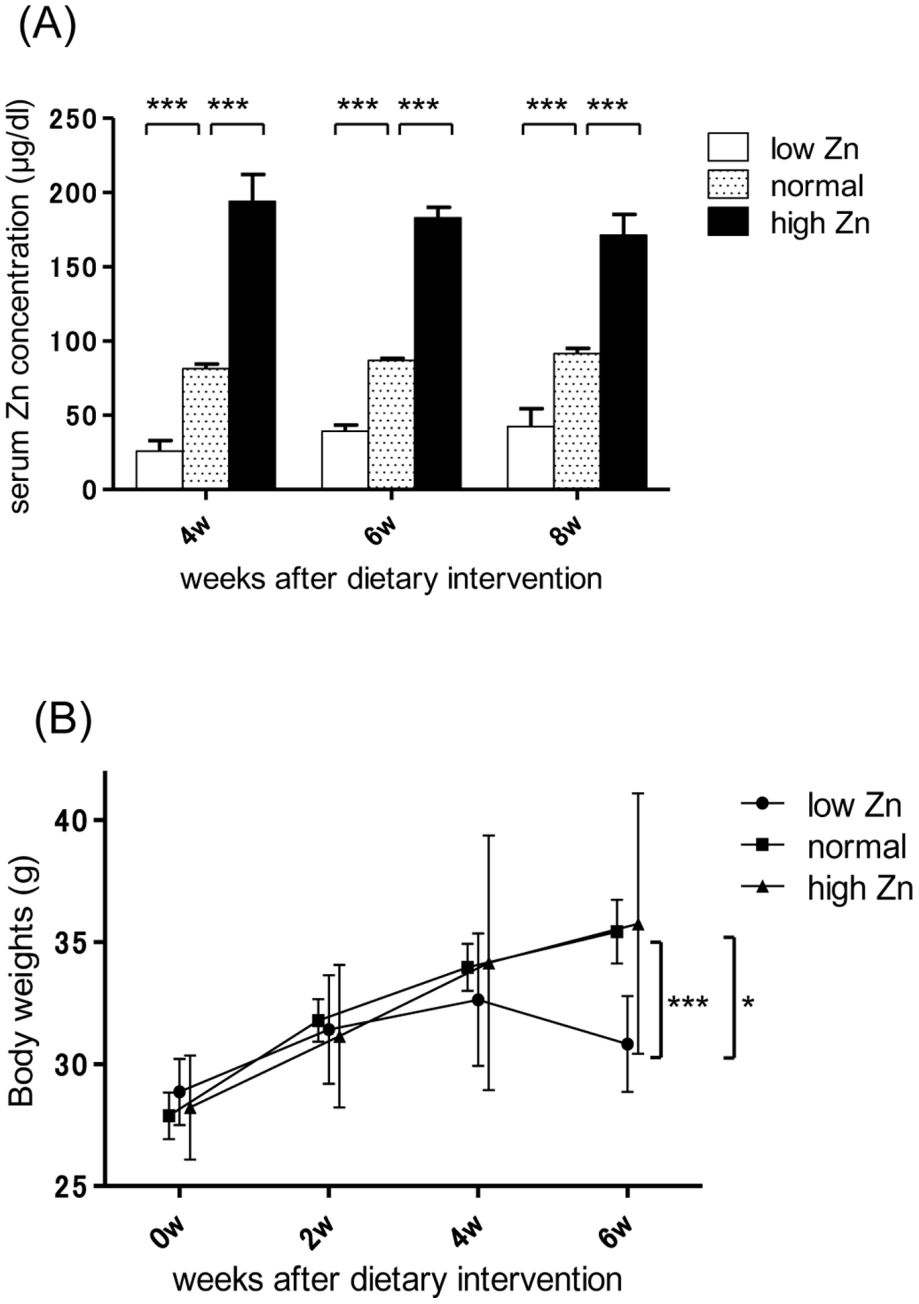
Physiological effect of dietary zinc intervention. (A) Changes in serum zinc (Zn) concentrations. Serum Zn concentrations were significantly different in each group from the fourth week after dietary intervention. (B) Changes in body weights. Significant body weight gain was suppressed in the low Zn diet group. *p<0.05; ***p<0.001.

Serum Zn concentrations significantly differed between the groups from the fourth week after dietary intervention (Figure 1A). As expected, body weight gain was suppressed in the low Zn diet group because of growth retardation caused by the Zn deficiency (Figure 1B). Symptoms such as diarrhea were not caused by the high Zn diet. Liver dysfunction was not observed in any dietary group at the 6th week. To avoid various redundant negative effects of a long-term Zn deficiency, such as growth retardation or severe immunodeficiency, we evaluated IgAN activity in the sixth week after intervention.

### Dietary Zinc Affects the Severity of Murine IgAN

**Figure 2:**
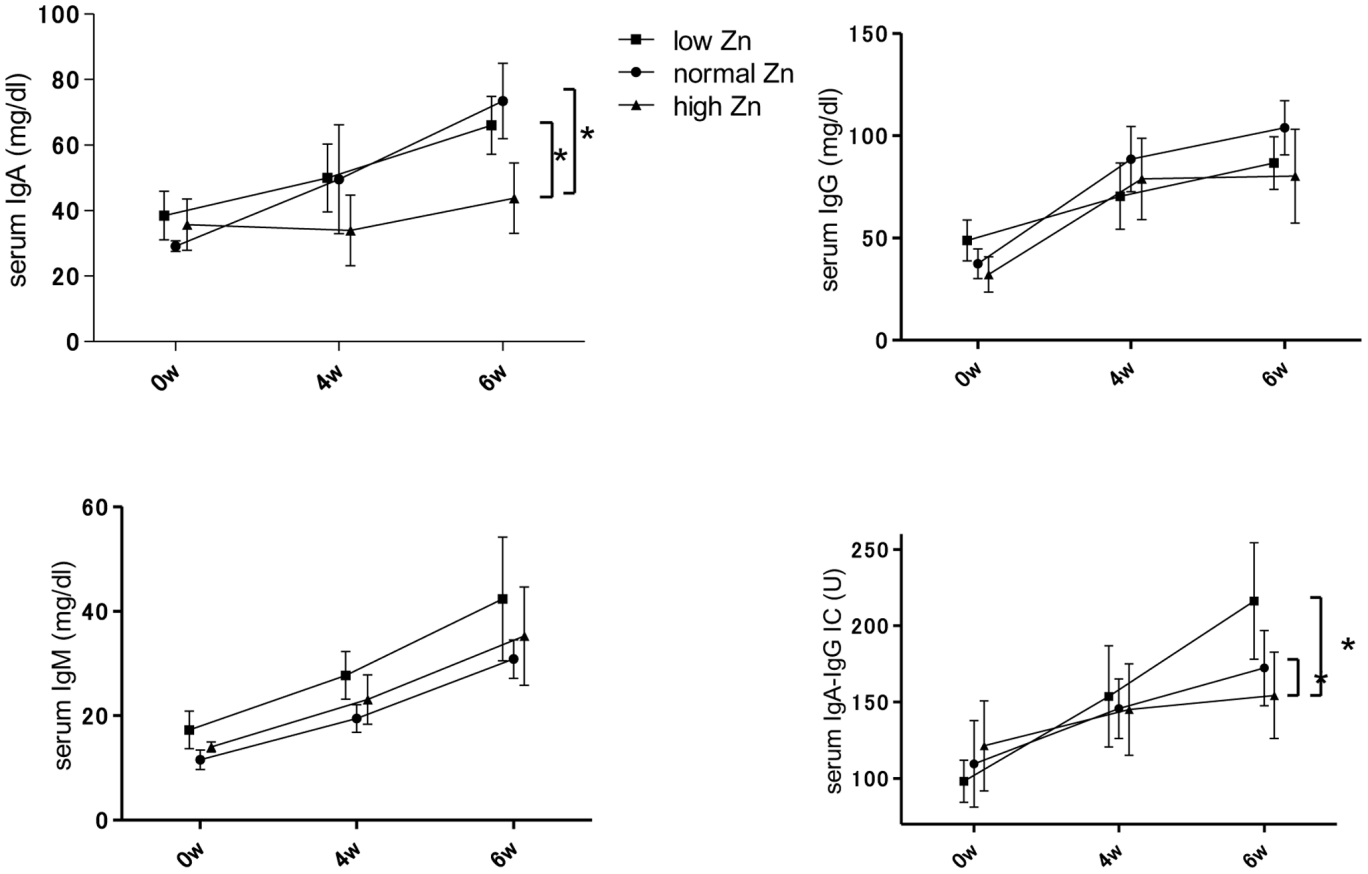
Serum concentration of immunoglobulins for each dietary zinc condition. Dietary zinc intervention altered the serum concentrations of IgA and IgA–IgG immune complexes but not of IgG and IgM. *p<0.05.

The number of splenic B cells was not different among each group. However, the serum concentrations of IgG, IgA, IgM, and IgA–IgG ICs in the low and normal Zn diet groups increased during the course of the disease. On the other hand, a significant suppression of serum IgA (p<0.05) and IgA–IgG ICs (p<0.05) was observed in the high Zn diet group in the sixth week (Figure 2).

**Figure 3:**
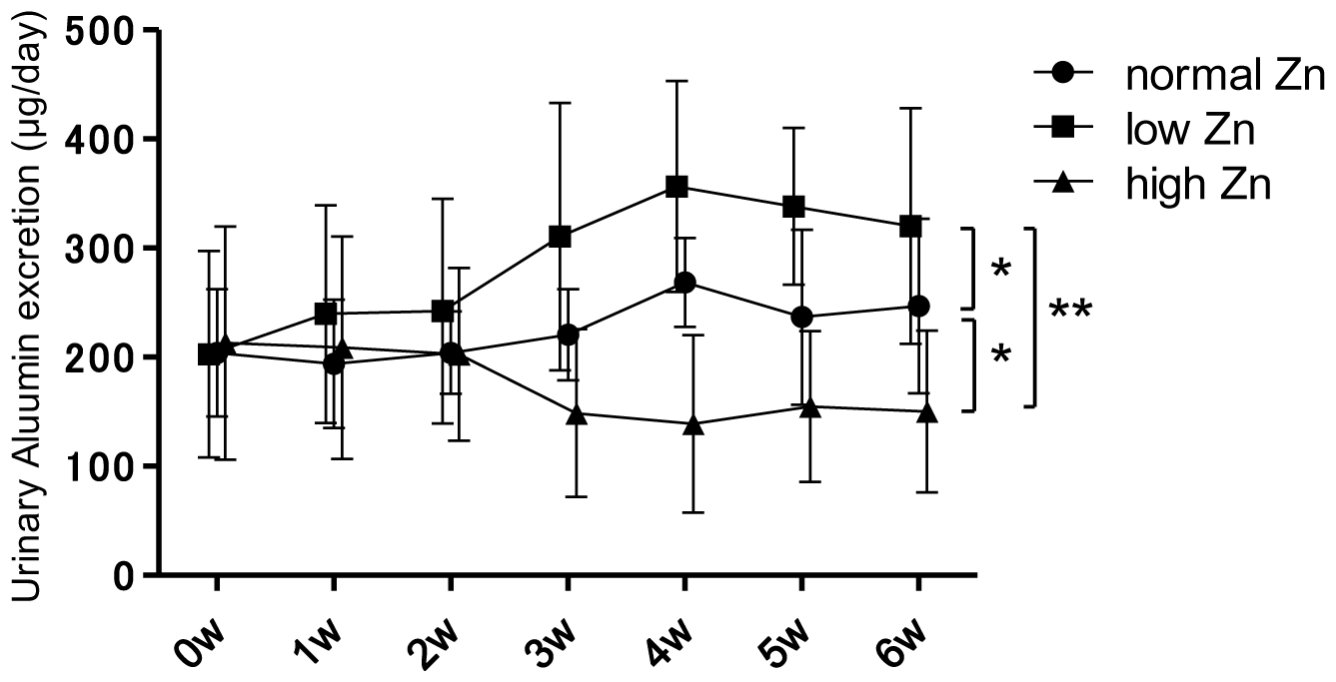
Urinary albumin levels for each dietary zinc condition. Urinary levels of albumin were significantly higher in the low zinc (Zn) diet group and lower in the high Zn diet group on the sixth week. *p<0.05; **p<0.01.

**Figure 4:**
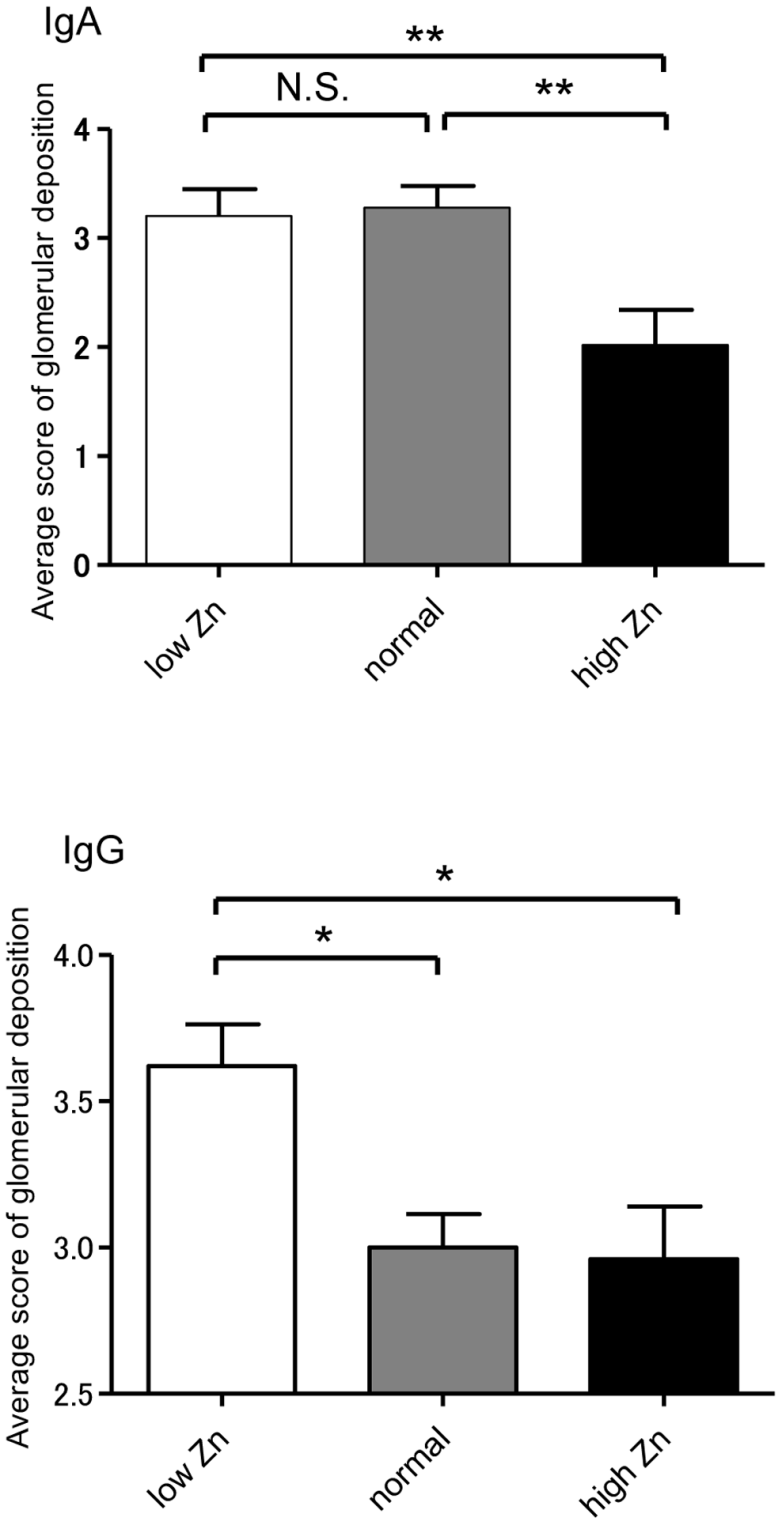
Intensity of mesangial IgA and IgG deposition. The intensity of IgA and IgG deposition was scored on a 1–5 scale based on an average of more than 10 glomeruli. IgA deposition was significantly attenuated in the high zinc (Zn) diet group. IgG deposition in the low Zn diet group was significantly higher than that in the normal and high Zn diet groups. *p<0.05; **p<0.01.

Urinary albumin levels in the low Zn diet group were significantly higher than that in the normal and high Zn diet groups (vs. normal Zn diet group: p<0.05; vs. high Zn diet group: p<0.01) in the sixth week (Figure 3). The intensity of glomerular IgA deposition in the high Zn diet group in the sixth week was significantly lower than that in the other groups (vs. normal Zn diet group: p<0.01; vs. low Zn diet group: p<0.01). In contrast, the low Zn diet group exhibited significantly higher IgG deposition in the glomeruli than the other groups (vs. normal Zn diet group: p<0.05; vs. high Zn diet group: p<0.05; Figure 4).

### Toll-like Receptor 4 Stimulation Accelerates Murine IgAN Progression

**Figure 5:**
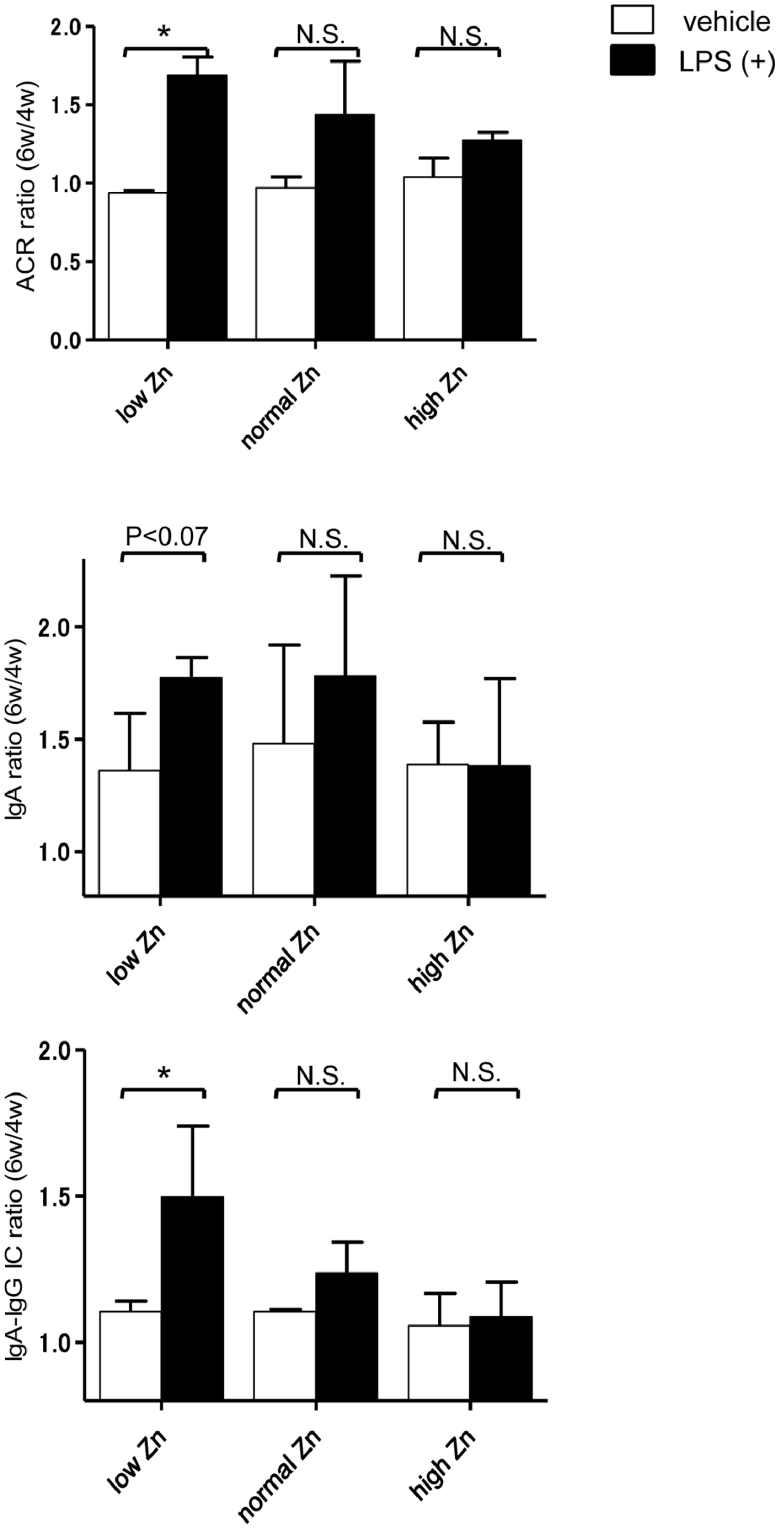
Reactivity against lipopolysaccharide. Lipopolysaccharide (LPS) was nasally administered to some mice in each dietary group, while others received saline as a vehicle control; samples were collected during the sixth week. We calculated the ratio (sixth:fourth week) of each parameter. Although ACR in all groups increased after LPS stimulation, the elevation in the low zinc (Zn) diet group was very high. Serum concentrations of IgA and IgA–IgG immune complexes (ICs) in the low and normal Zn diet groups tended to be higher after LPS stimulation. However, the high Zn diet group maintained low concentrations of serum IgA and IgA–IgG ICs despite LPS stimulation. *p<0.05.

To investigate whether dietary Zn levels influenced the mucosal TLR-mediated aggravation of IgAN, we performed nasal challenges with CpG DNA and LPS in each dietary group. The effect of the nasal challenge with CpG DNA against serum immunoglobulins (IgG, IgA, IgM, and IgA–IgG ICs) and urinary albumin were not different in each dietary group (data not shown). Although all mice in each dietary group showed an aggravation of IgAN after nasal LPS stimulation, increased albuminuria was more pronounced in the low Zn diet group (p<0.05), with a greater increase in serum concentrations of IgA (p<0.07) and IgA–IgG ICs (p<0.05; Figure 5). In contrast, the changes in the serum concentrations of IgA and IgA–IgG ICs after LPS stimulation was clearly suppressed in the high Zn diet group. These results suggest that dietary Zn levels influence the TLR4-mediated progression of murine IgAN.

### Zinc Regulation Affects IgA Production by Spleen Cells

**Figure 6:**
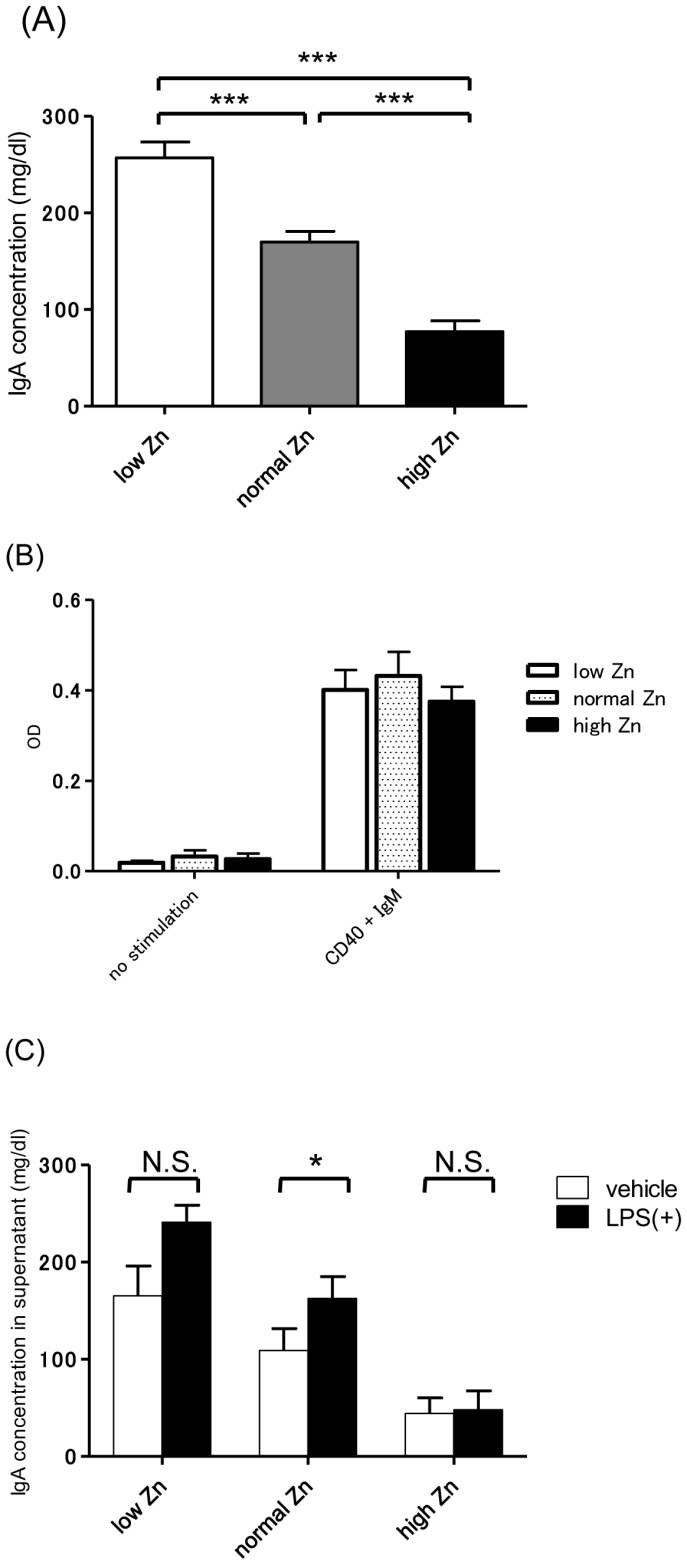
IgA production by whole spleen cells for each zinc condition. (A) Production of IgA was significantly higher in the low zinc (Zn) diet group and lower in the high Zn diet group. (B) The viability of splenic B cells in each Zn condition was not significantly different. (C) Reactivity against lipopolysaccharide (LPS) in each Zn condition. In the normal and low Zn diet groups, IgA production tended to be higher in the presence of LPS. However, in the high Zn diet group, serum IgA concentration remained at a low level despite of the presence of LPS. *p<0.05; ***p<0.001.

To further assess the association between dietary Zn levels and IgA production, we examined IgA production by spleen cells for each Zn level. In the absence of LPS, IgA production by spleen cells in low Zn conditions was significantly higher than those under normal (p<0.001) and high (p<0.001) Zn conditions (Figure 6A). Furthermore, these different levels of IgA production in each Zn condition were independent of the viability of B cells (Figure 6B). Under normal and low Zn conditions, IgA production tended to be higher in the presence of LPS. However, under high Zn conditions, serum IgA concentration remained at a low level despite the presence of LPS (Figure 6C).

**Figure 7:**
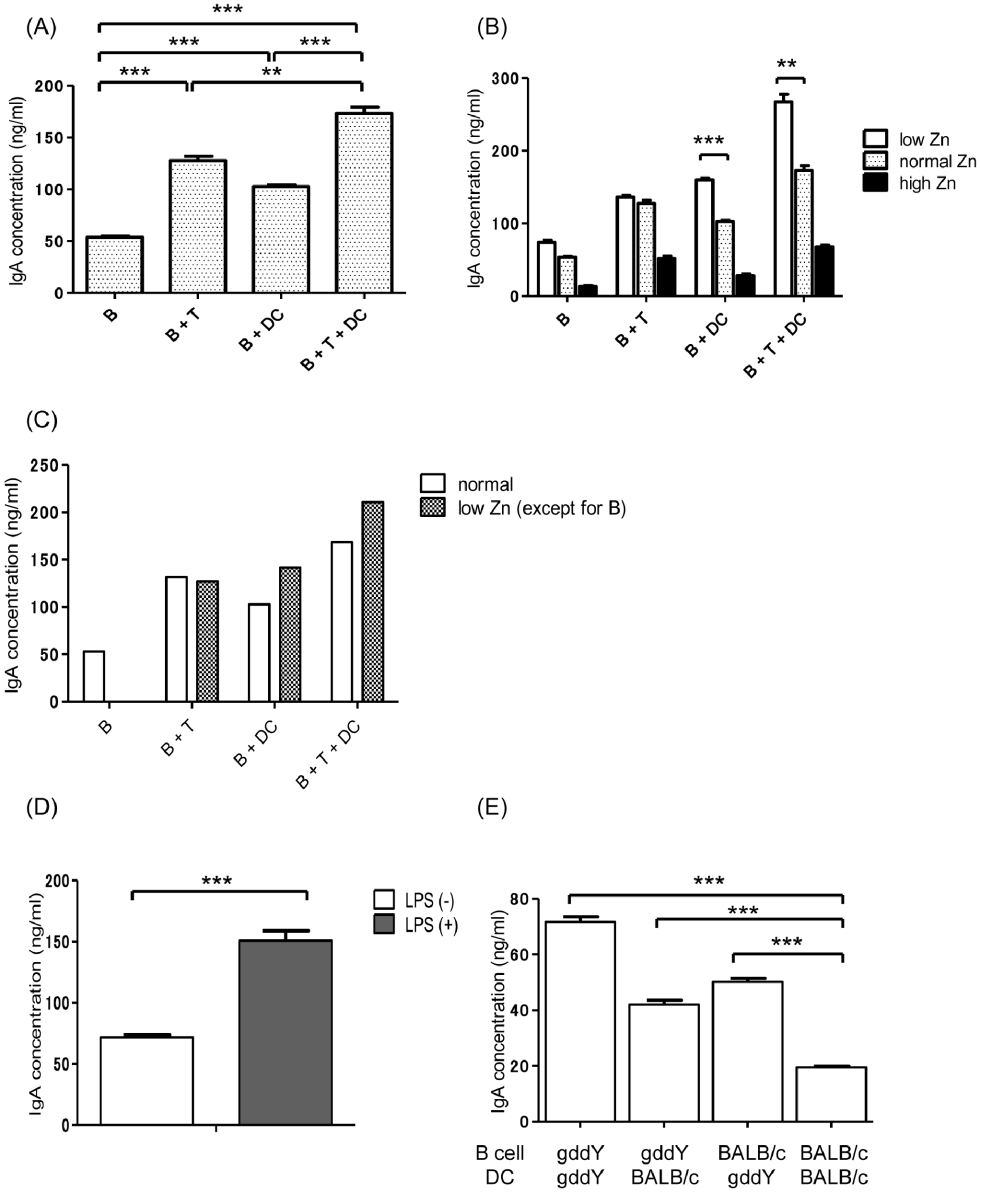
IgA production by various spleen cells. (A) IgA production by B cells increased when cocultured with T cells and/or dendritic cells (DCs). (B) Under low zinc (Zn) conditions, IgA production was significantly increased in the presence of DCs. Under high Zn conditions, IgA production by all cell combinations was suppressed. (C) Coculture of untreated B cells with low Zn-treated T cells and/or DCs. IgA production increased in the presence of DCs. (D) Coculture of B cells and DCs with or without lipopolysaccharide (LPS). LPS significantly enhanced IgA production despite the absence of T cells. (E) Coculture of B cells and DCs from gddY or BALB/c mice. A combination of both B cells and DCs from gddY mice yielded the highest production of IgA. **p<0.01; ***p<0.001.

B cells, T cells, and DCs were isolated from spleen cells to further investigate the influence of Zn on each cell type and their interactions. As expected, IgA production by B cells increased when cocultured with T cells and/or DCs (Figure 7A). Under low Zn conditions, IgA production was significantly increased in the presence of DCs, suggesting that elevated IgA production under low Zn conditions is mainly mediated by B cell – DC interaction (Figure 7B). In contrast, under high Zn conditions, IgA production was suppressed in all cell combinations (Figure 7B). Similar results were evident when we cultured untreated B cells with T cells and/or DCs from low Zn conditions (Figure 7C). In addition, LPS significantly enhanced IgA production when cocultured with DCs (p<0.001), confirming that DCs activated by LPS directly increase IgA production in a T cell-independent manner (Figure 7D). However, when we cocultured B cells and DCs from gddY or BALB/c mice, a combination of both B cells and DCs from gddY mice showed the highest levels of IgA production (Figure 7E). Briefly, the production of nephritogenic IgA by gddY mice is due to a functional abnormality of both B cells and DCs.

**Figure 8:**
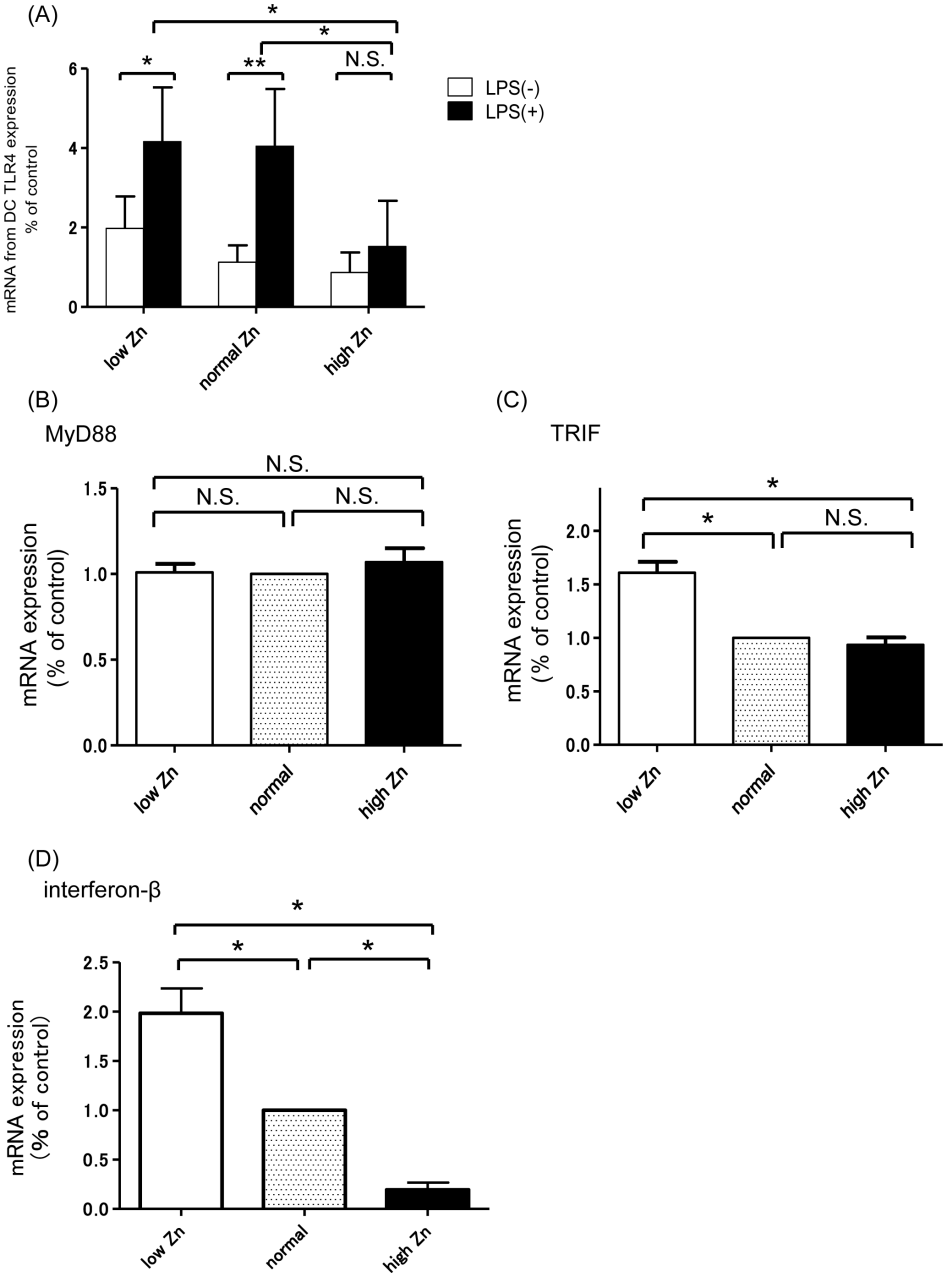
Expression of Toll-like receptor 4, MyD88, TRIF, and interferon-β in each zinc condition. (A) Expression of Toll-like receptor 4 (TLR4) for each zinc (Zn) condition with/without lipopolysaccharide (LPS). Expression of TLR4 was significantly increased when stimulated by LPS under low and normal Zn conditions. However, TLR4 expression under high Zn conditions was low despite LPS stimulation. (B) Expression of MyD88 at each Zn condition. Expression of MyD88 remained constant at each Zn condition. (C) Expression of TRIF for each Zn condition. Expression of TRIF under low Zn conditions was significantly higher than that in other groups. (D) Expression of interferon-β in each Zn condition. Expression of interferon-β was higher in low Zn condition and lower in high Zn condition. *p<0.05; **p<0.01.

Expression levels of TLR4 and TLR9 in DCs for each Zn level were checked with or without LPS stimulation. The expression of TLR9 was unaffected by Zn or LPS stimulation (data not shown). In contrast, the expression of TLR4 significantly increased under LPS stimulation under low (p<0.05) and normal (p<0.01) Zn conditions. However, TLR4 expression under high Zn conditions was attenuated, even in the presence of LPS (vs. normal Zn diet group: p<0.05; vs. low Zn diet group: p<0.05; Figure 8A). We also examined the expression of MyD88 and TIR-domain-containing adapter-inducing interferon-β (TRIF), which are key signaling molecules of TLR4. Although the expression of MyD88 was unaffected by Zn (Figure 8B), the expression of TRIF under low Zn conditions was significantly upregulated (vs. normal Zn diet group: p<0.05; vs. high Zn diet group: p<0.05; Figure 8C). To confirm the functional alteration of the TLR4/TRIF pathway caused by Zn, we examined the expression of interferon-β, which is specifically induced by the activation of the TRIF-dependent pathway following TLR4 activation [28], [29]. The expression of interferon-β was higher in low Zn condition and lower in high Zn condition. (Figure 8D).

## Discussion

Environmental factors are considered to have an impact on susceptibility to IgAN. Johnson et al. [7] assessed the geographic bias in the prevalence, from the perspective of the immunological effects of exposure to exogenous antigens. Moreover, diet may be involved in the pathogenesis of IgAN, as well as in other atopic diseases [30] considered to be associated with hygiene. As dietary intervention is easy and has few adverse effects, clarifying the association between diet and such diseases through dietary intervention may represent an important step toward new potential treatments for IgAN. However, few reports, except for a small number on fish oil [11] and gluten [5], discuss the connection of diet and renal diseases. It is difficult to identify one key factor among the vast number of nutritive components involved in diet. In this study, we focused on the immunological functions of Zn, an important component of the immune system, and investigated the relationship between dietary Zn levels and IgAN development. We discovered that the dietary Zn levels are critically involved in the immune process of nephritogenic IgA production by affecting the activity of DCs, especially through the TLR4/TRIF pathway.

We used IgAN-prone gddY mice, which exhibit the same renal phenotype and dysregulation of the systemic immune system as patients with IgAN. We recently demonstrated that the renal prognosis of gddY mice may be linked to differences in sugar content or the O-glycosylation acceptor site of the IgA hinge region [25]. These findings suggest that the progression of this murine IgAN also may be dependent on the aberrantly glycosylated IgA, similar to that seen in human IgAN. We previously reported that, in ddY mice under conventional conditions the serum concentrations of IgA, but not those of IgG and IgM, were significantly elevated relative to mice maintained under specific pathogen-free (SPF) conditions [8]. This study showed that Zn supplementation suppressed urinary albumin levels in association with a decrease in the serum concentrations of IgA and IgA–IgG ICs, but not those of IgG and IgM. These findings led us to conclude that Zn-mediated mechanisms are specifically involved in the increased nephritogenic IgA production in response to mucosal exogenous antigens. In fact, the dietary Zn supplementation reduced mesangial IgA deposition, in association with decreasing concentrations of IgA and IgA–IgG ICs, even after mucosal LPS stimulation. In contrast, low Zn levels worsened the course of the disease with or without LPS. These results suggest that the dietary Zn levels influence the mucosal immune response, including nephritogenic IgA production.

The *in vitro* study confirmed the *in vivo* results: IgA production by splenocytes was increased at low Zn levels and decreased at high Zn levels. Interestingly, the influence of Zn on IgA production was abrogated in the absence of DCs. To avoid a direct effect of Zn on B cells, we cocultured untreated B cells with Zn-treated T cells and/or DCs and obtained similar results. These findings indicate that Zn regulation is associated with an immune reaction mediated by DCs. On the other hand, DCs from gddY mice played a critical role in IgA production. The concentration of IgA production by B cells was higher when cocultured with DCs, irrespective of the presence of T cells. Similarly, in a T cell-independent manner, DCs stimulated by LPS directly altered the IgA production by B cells (Figure 7D). It was also evident that DCs from gddY mice were capable of inducing higher levels of IgA production by B cells than the same number of DCs from BALB/c mice, even when cultured under the same conditions (Figure 7E), suggesting that DCs from gddY mice have an increased capacity to induce IgA production than those from BALB/c mice.

Next, we analyzed the effect of Zn and exogenous antigens on DCs. We previously reported that the activation of the TLR9/MyD88 pathway on B cells or DCs is a critical determinant of IgAN severity [8], [31]. However, this study revealed that Zn affects the reactivity of TLR4, but not that of TLR9. Thus, we assessed the expression of TLR4 in splenic DCs under various Zn and LPS conditions. Although LPS stimulation increased TLR4 expression by DCs under normal and low Zn conditions, there was little change in TLR4 expression by DCs under high Zn conditions, irrespective of LPS stimulation. This result is consistent with a previous report demonstrating that Zn homeostasis is involved in some DC maturation events and affects the magnitude of immune responses via the activation of TLR4 [22].

Each TLR triggers a specific biological response. MyD88 is universally used by all TLRs except TLR3, and induces inflammatory cytokines. In contrast, TRIF is used by TLR3 and TLR4 only. TLR4 is therefore the only TLR that activates both the MyD88 and TRIF signaling pathways [29]. We investigated the expression of these two signaling molecules. Although MyD88 expression was unaltered at each Zn level, TRIF expression was significantly higher at low Zn levels. MyD88 induces inflammatory responses by activating NF-κB and MAPK, whereas TRIF recruits TRADD, TRAF6, and TRAF3. TRADD and TRAF6 also lead to activation of MAPK and NF-κB. On the other hand, TRAF3 activates some kinases, such as TBK1 and IKKi, and subsequently, IRF3, which is a key transcriptional factor for interferon-β [29]. Therefore, during TLR4 activation, interferon-β is specifically induced by the TRIF signaling pathway and not by the MyD88 signaling pathway. The present study showed that expression levels of interferon-β and TRIF were both enhanced in low Zn conditions, indicating the involvement of Zn in the TRIF pathway. Furthermore, although expression levels of TRIF were not significantly different, expression levels of interferon-β in high Zn conditions were lower than those in normal Zn conditions. These data suggest that a high Zn condition may directly attenuate the activation of the downstream TRIF signaling pathway rather than TRIF expression itself. These findings are consistent with the *in vivo* and other *in vitro* experiments, and therefore, provide further support for the Zn effect on IgA production via the TLR4/TRIF pathway. In summary, we demonstrated that Zn alters nephritogenic IgA production and, thus, is critically involved in disease activity in murine IgAN. These findings suggest that diet, as a major environmental factor, plays an important role in the pathogenesis of IgAN, as well as in other immune-related diseases. Zn modulates IgA production by altering the activity of DCs, especially via TLR4/TRIF. In other words, dietary Zn levels may determine the susceptibility to environmental factors, such as exogenous antigens. As a recent study demonstrated the clinical application of Zn supplements combined with other supplementation in immune-related diseases, including asthma, Zn supplementation represents a therapeutic strategy for preventing the progression or aggravation of IgAN [32].
